# Time to appropriate antibiotic therapy is a predictor of outcome in patients with bloodstream infection caused by KPC-producing *Klebsiella pneumoniae*

**DOI:** 10.1186/s13054-020-2742-9

**Published:** 2020-01-30

**Authors:** Marco Falcone, Matteo Bassetti, Giusy Tiseo, Cesira Giordano, Elia Nencini, Alessandro Russo, Elena Graziano, Enrico Tagliaferri, Alessandro Leonildi, Simona Barnini, Alessio Farcomeni, Francesco Menichetti

**Affiliations:** 1 Infectious Diseases Unit, Azienda Ospedaliera Universitaria Pisana, University of Pisa, Via Paradisa, 2, 56124 Pisa, PI Italy; 2 Infectious Diseases Clinic Department of Health Science, University of Genoa and Hospital Policlinico San Martino – IRCCS, Genoa, Italy; 3 Microbiology Unit, Azienda Ospedaliera Universitaria Pisana, Pisa, Italy; 4 Emergency Medicine Department, University of Pisa, Pisa, Italy; 5 Infectious Diseases Division, Department of Medicine, University of Udine and Azienda Sanitaria Universitaria Integrata di Udine, Udine, Italy; 6 Department of Economics and Finance, University of Rome “Tor Vergata”, Rome, Italy

**Keywords:** Bloodstream infections, Bacteremia, Antibiotic resistance, Carbapenem-resistant, Carbapenemases, KPC, *Klebsiella pneumoniae*, Mortality, Time to appropriate antibiotic therapy

## Abstract

**Background:**

Bloodstream infections (BSIs) by *Klebsiella pneumoniae* carbapenemase (KPC)-producing *Klebsiella pneumoniae* (Kp) are associated with high mortality. The aim of this study is to assess the relationship between time to administration of appropriate antibiotic therapy and the outcome of patients with BSI due to KPC-Kp hospitalized in intensive care unit (ICU).

**Methods:**

An observational study was conducted in the ICUs of two academic centers in Italy. Patients with KPC-Kp bacteremia hospitalized between January 2015 to December 2018 were included. The primary outcome was the relationship between time from blood cultures (BC) collection to appropriate antibiotic therapy and 30-day mortality. The secondary outcome was to evaluate the association of different treatment regimens with 30-day mortality and a composite endpoint (30-day mortality or nephrotoxicity). A Cox regression analysis to identify factors independently associated with 30-day mortality was performed. Hazard ratio (HR) and 95% confidence interval (CI) were calculated.

**Results:**

A total of 102 patients with KPC-Kp BSI were included. The most common sources of infection were intra-abdominal (23.5%), urinary tract (20.6%), and skin and skin structure (17.6%). The 30-day mortality was 45%. Median time to appropriate antibiotic therapy was shorter in patients who survived (8.5 h [IQR 1–36]) versus those who died (48 h [IQR 5–108], *p* = 0.014). A propensity score matching showed that receipt of an in vitro active therapy within 24 h from BC collection was associated with lower 30-day mortality (HR = 0.36, 95% CI: 0.188–0.690, *p* = 0.0021). At Cox regression analysis, factors associated with 30-day mortality were primary bacteremia (HR 2.662 [95% CI 1.118–6.336], *p* = 0.027), cardiovascular disease (HR 2.196 [95% CI 1.082–4.457], *p* = 0.029), time (24-h increments) from BC collection to appropriate therapy (HR 1.382 [95% CI 1.132–1.687], *p* = 0.001), SOFA score (HR 1.122 [95% CI 1.036–1.216], *p* = 0.005), and age (HR 1.030 [95% CI 1.006–1.054], *p* = 0.012). Ceftazidime-avibactam-containing regimens were associated with reduced risk of composite endpoint (30-day mortality OR nephrotoxicity) (HR 0.231 [95% CI 0.071–0.745], *p* = 0.014) compared to colistin-containing regimens.

**Conclusions:**

Time to appropriate antibiotic therapy is an independent predictor of 30-day mortality in patients with KPC-Kp BSI. Appropriate antibiotic therapy should begin within 24 h from the collection of BC.

**Electronic supplementary material:**

The online version of this article (10.1186/s13054-020-2742-9) contains supplementary material, which is available to authorized users.

## Introduction

The spread of *Klebsiella pneumoniae* carbapenemase (KPC)-producing *Klebsiella pneumoniae* (Kp) is an urgent public health threat requiring immediate action [1, 2]. As a matter of fact, carbapenem-resistant (CR) Kp infections are associated with high attributable mortality (2118 attributable deaths out of a total of 15,947 infections), and with a median number of total disability-adjusted life years of 11.5 per 100,000 population [3]. Mortality rates of patients with bloodstream infections (BSIs) caused by KPC-Kp range from 40 to 50% [4, 5].

Treatment of patients with BSIs caused by KPC-Kp represents a challenge for clinicians, and no data from randomized clinical trials comparing different antibiotic strategies are currently available [6]. Observational studies indicate that combination therapies are more effective than monotherapies [7, 8]. Moreover, a retrospective study performed in intensive care unit (ICU) patients with septic shock showed that an antibiotic regimen containing at least two in vitro active antibiotics is associated with improved survival [9]. Novel drugs represent new promising treatment options, but larger studies are needed to evaluate their impact on survival of patients with KPC-Kp infections [10–12]. Thus, the optimal therapy of KPC-Kp BSI has not been defined.

Timely and appropriate antimicrobial therapy is critically important for treatment of patients with BSI [13, 14]. Recent studies highlighted that inappropriate empiric treatment is associated with poor outcome of patients with urinary tract [15] or any type of infection due to KPC-Kp [16, 17]. Among non-neutropenic patients with BSI due to KPC-Kp, Satlin et al. did not find any association between timing of active therapy and mortality, but a trend to higher mortality rates was observed in patients who did not receive active therapy within the first 12 h from BSI onset [18]; however, the majority of patients (84%) acquired infection in the community or in non-ICU wards [18]. Thus, the relationship between timing of appropriate therapy and outcome of BSI caused by KPC-Kp has not been specifically investigated in ICU patients.

The aim of this study was to assess the relationship between the time to appropriate antibiotic therapy and the clinical outcome in ICU patients with BSI caused by KPC-Kp.

## Methods

### Study design and data collection

This observational retrospective study was conducted from January 2015 to December 2018 in two University tertiary care hospitals: Azienda Ospedaliera Universitaria Pisana (Pisa, Italy) and Santa Maria della Misericordia Hospital (Udine, Italy). All consecutive adult patients with a BSI due to KPC-Kp hospitalized in ICU were included in the study. Patients with polymicrobial BSIs were excluded. The study was conducted according to the principles stated in the Declaration of Helsinki.

Data were extracted from the medical records of patients and from hospital computerized databases. The following data were collected: demographics, clinical and laboratory findings, comorbidities, microbiologic data, source of infection, and source control. All patients underwent screening rectal swab at admission in the ICU and twice weekly. Data about rectal colonization by KPC-Kp were recorded. Sequential Organ Failure Assessment (SOFA) score at time of BSI and APACHE II score were calculated [19, 20]. Data about hospitalization and antibiotic therapy in the previous 90 days before the BSI were also reviewed. Treatment regimens were analyzed and time from collection of blood cultures to administration of appropriate antibiotic therapy was calculated and reported in hours.

### Outcome and assessments

The primary outcome of the study was the relationship between the time to appropriate antibiotic therapy and 30-day mortality. Time to appropriate antibiotic therapy was defined as the time (in hours) from blood culture collection and the administration of in vitro active antibiotic therapy. In order to evaluate the impact of time to appropriate antibiotic therapy on 30-day mortality, time was assessed as categories of 24-h increments.

The secondary outcome was to evaluate if different treatment regimens influence the 30-day mortality and a composite endpoint of mortality or nephrotoxicity (post-baseline increase in serum creatinine > 1.0 mg/dL or adverse events preferred term of renal failure, renal failure acute, or renal impairment). To this end, we identified three groups of treatment: (1) colistin-containing regimen, (2) ceftazidime/avibactam (± fosfomycin or aminoglycosides), (3) other regimens.

### Definitions

A KPC-Kp BSI was defined as a BSI documented by blood culture positivity for a KPC-Kp strain. According to the National Healthcare Safety Network, the probable source of BSI was assessed according to the available clinical and microbiological information and classified by the investigators using the following categories: BSI from central venous catheter (CVC), respiratory tract infection, urinary tract infection (UTI), skin and skin structure infection (SSTI), intra-abdominal infection (IAI), endocarditis, and primary BSI in the absence of an identified source of infection growing the same organism as recovered from blood [21]. The CVC was considered a likely source of infection if the blood culture obtained from the lumen of the catheter was positive at a time < 2 h compared to peripheral veins, and/or culture of the catheter was positive.

BSI onset was defined as the date of collection of the index blood culture. Time to adequate antibiotic therapy was defined as the number of hours from blood culture collection and the administration of active antibiotic therapy. Control of removable source of infection was defined as removal of any preexisting contaminated CVC and as drainage of intra-abdominal abscesses or other fluid collections thought to be the source of KPC-Kp infection within 24 h from the onset of BSI. Cardiovascular disease was defined as the presence of one or more of the following conditions: previous coronary heart disease, peripheral arterial disease, heart failure, paroxysmal atrial fibrillation, and chronic (persistent or permanent) atrial fibrillation [22].

BSI episodes were classified as nosocomial, healthcare-associated, and community-acquired according to previous definitions [23]. Nosocomial BSIs were defined by positive blood cultures obtained from patients who had been hospitalized for 48 h or longer; health care-associated BSIs were defined by positive blood cultures obtained from a patient at the time of hospital admission or within 48 h of admission if the patient fulfilled any of the following criteria: received intravenous therapy at home, received wound care or specialized nursing care or had self-administered intravenous medical therapy in the 30 days before the BSI, attended a hospital or hemodialysis clinic or received intravenous chemotherapy in the 30 days before the BSI, was hospitalized in an acute care hospital for two or more days in the 90 days before the BSI, or was a resident in a nursing home or long-term care facility. Community-acquired BSI was defined by a positive blood culture obtained at the time of hospital admission or within the 48 h after hospital admission for patients who did not fit the criteria for a health care-associated infection [23]. The presence of septic shock was defined according to the last proposed criteria (Sepsis-3) [24]. Information about specific causes of death was collected. Cause of death was defined as the primary pathology (irrespective of its duration) leading to death of the patient and was classified as previously reported [25].

### Antimicrobial treatment evaluation

All patients were evaluated by a dedicated infectious disease specialist throughout their entire ICU hospitalization. Empiric antibiotic therapy was selected according to clinical judgment by infectious disease specialists, which was subsequently modified according to blood cultures results. According to internal ICU protocols, tigecycline was administered at high dosages (100 mg iv q12h), and ß-lactams, ß-lactamase inhibitors, and carbapenems were administered using extended infusion (meropenem over 4–6 h). According to local ICU protocols, colistin dosages were uniformly administered at the dosages of 9 million IU daily in patients with normal renal function. Modification of dosage schedules based on renal function was performed according to indications from the Food and Drug Administration [26].

Antibiotic treatment was classified as appropriate if at least one administered antibiotic displayed documented in vitro susceptibility according to the breakpoints established by the European Committee on Antimicrobial Susceptibility Testing (EUCAST). Combination therapy was defined as treatment regimens including two or more in vitro active antibiotics, administered at the same time. Antibiotic regimens were also analyzed according to the following classification: no antibiotic displaying in vitro activity, only one antibiotic displaying in vitro activity, and two or more antibiotics displaying in vitro activity.

### Microbiology and KPC identification

From positive blood cultures, Gram staining and a rapid identification protocol were adopted [27]. The bacterial pellet obtained directly from positive blood cultures was used for MALDI-TOF MS (Bruker Daltonics) identification and for molecular analysis. The presence of a bla_KPC_ gene was determined by PCR using the GeneXpert® System (Cepheid). Antimicrobial susceptibility tests were performed with the SensiTitre™ system (Thermo Fisher Scientific) or Vitek 2 automated system (bioMérieux, Marcy l’Etoile, France) according to the manufacturer’s instructions. Minimum inhibitory concentrations (MICs) were classified according to breakpoints established by the European Committee on Antimicrobial Susceptibility Testing (EUCAST) [28].

### Statistical analysis

Continuous variables were reported as mean ± standard deviation and medians and interquartile ranges (IQRs) according to their distribution. The Student *t* test was used when comparing normally distributed data, and the Mann-Whitney *U* test was used to analyze non-normally distributed data. Categorical data were expressed as frequency distributions, and the chi-square test or Fisher exact test was used to determine if differences existed between groups.

According to the primary outcome of the study, a Cox regression analysis was performed to determine the effects of different variables on the overall 30-day mortality and on the 30-day mortality specifically due to infection. Multivariate analysis using logistic regression prediction models was constructed using a forward stepwise procedure, entering all variables with univariate *p* < 0.05 and those deemed clinically significant. Age, SOFA score, and time from blood culture collection to appropriate antibiotic therapy were continuous (24-h increments were considered for time to appropriate therapy). Potential baseline confounders, including diabetes, cardiovascular disease, COPD, and primary bacteremia were considered for the multivariate analysis. The final multivariate model was chosen according to the Akaike information criterion and to parsimony and clinical interpretability of data. Kaplan-Meier curves were constructed to compare time to 30-day mortality between patients who received appropriate antibiotic therapy within the first 24 h from the blood cultures collection and those who did not. Moreover, a more formal causal analysis was conducted based on propensity scores (PS). Propensity scores were estimated using a logistic regression model to predict time to adequate therapy < 24 h using age, diabetes, Charlson Comorbidity index, and SOFA score. Balance tables were used to assess adequacy of matching via propensity score. Estimated propensity scores were then used to perform greedy matching without replacement. The matched subset was then used to estimate the relationship between time to adequate therapy and the endpoints using Cox regression. We evaluated both univariate Cox regression models and models including a random effect specific to each matched couple (conditional Cox regression), whose results did not differ substantially.

A sensitivity analysis was performed to evaluate the effect of combination therapy (defined as above-reported) on 30-day mortality. A forest plot was used to describe the results of the sensitivity analysis.

To evaluate differences in clinical outcome among treatment regimens, differences in 30-day mortality and in the composite endpoint of 30-day mortality or nephrotoxicity were analyzed using the Cox analysis. Colistin-containing regimen was selected as reference variable; both ceftazidime-avibactam-containing regimen and other regimens were tested against the reference variable. Patients who did not receive any in vitro active therapy was excluded. Hazard ratio (HR) and 95% confidence intervals (CIs) were calculated to evaluate the strength of any association. Statistical significance was established at *p* ≤ 0.05. All reported *p* values are two tailed. The results obtained were analyzed using a commercially available statistical software package (SPSS 20.0; IBM, Armonk, NY, USA and R 3.5.1, Vienna, Austria).

## Results

### Study population

**Fig. 1:**
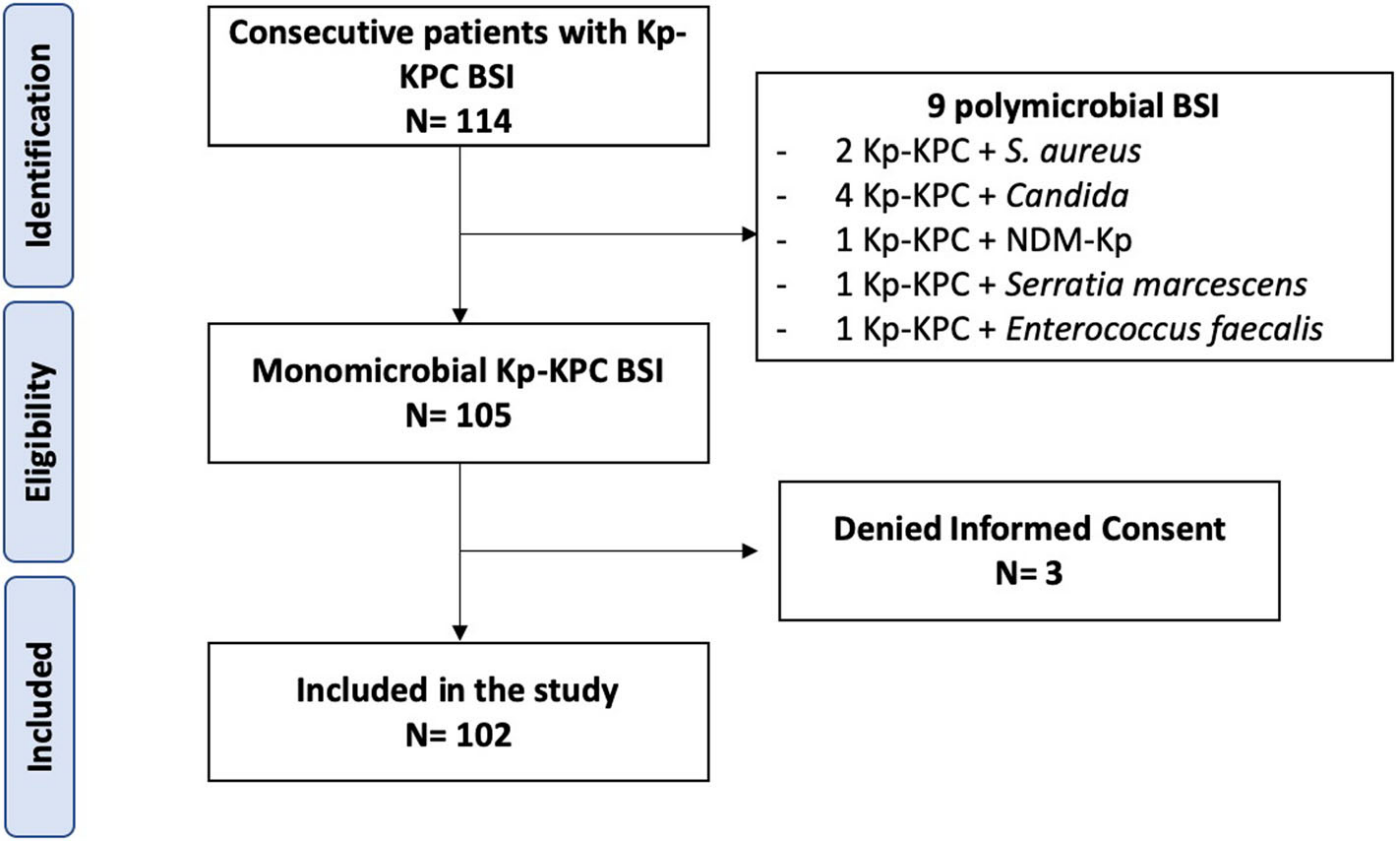
Study flow chart. BSI bloodstream infections, Kp-KPC *Klebsiella pneumoniae* carbapenemase-producing *Klebsiella pneumoniae*, NDM New-Delhi-metallo-beta-lactamase

Among 114 ICU patients with Kp-KPC BSI, a total of 102 were included in the study. Flowchart of the study is shown in Fig. 1. Overall, median age was 64 years (IQR 53–74). The most common source of infection was IAI (23.5%), followed by UTI (20.6%), SSTI (17.6%), CVC (12.7%), and pneumonia (10.8%). Fourteen (13.7%) patients had a primary BSI. The majority of patients had recorded previous rectal colonization by KPC-Kp (51%).

Septic shock occurred in 39.2% of patients. The 7-day, 14-day, and 30-day mortality rates were 20.6%, 32.4%, and 45%, respectively. All deaths occurred during the ICU stay. Among 46 non-surviving patients, 43 (93.5%) died due to infection, 2 patients (4.3%) from acute hemorrhage, and 1 (2.2%) from acute respiratory failure. The baseline characteristics of the study population and the univariate analysis of patients by survival are reported in Table 1**.** Compared to surviving patients, those who died had a higher median age (70.5 [IQR 60–77.25] vs 55 years [IQR 41.5–70.75], *p* < 0.001) were more likely to be affected by diabetes mellitus (28.3% vs 8.9%, *p* = 0.011), cardiovascular disease (54.3% vs 19.6%, *p* < 0.001), and chronic obstructive pulmonary disease (17.4% vs 3.6%, *p* = 0.020), and had a higher median SOFA score (7.5 [IQR 4–9.25] vs 4 [IQR 2–10], *p* = 0.034). CVC-related bacteremia was more frequent among survivors (19.6% vs 4.3%, *p* = 0.021), while a primary BSI was more common among patients who died (26.1% vs 3.6%, *p* = 0.001). Three patients of 87, 88, and 90 years old, respectively, with multiple comorbidities received a “do not resuscitate order (DNR); one of them had severe burn injury.

**Table 1:** Baseline characteristics of population and univariate analysis of patients with KPC-Kp BSI by survival status

Characteristic	All patients *N* = 102	Patients who survived * N*= 56	Patients who did not survive * N*= 46	*p* value
Age, years, median (IQR)	64 (53–74)	55 (41.5–70.75)	70.5 (60–77.25)	*< 0.001*
Male sex	65 (63.7%)	39 (69.6%)	26 (56.5%)	0.170
Comorbidities				
Diabetes	18 (17.6%)	5 (8.9%)	13 (28.3%)	*0.011*
Cardiovascular disease	36 (35.3%)	11 (19.6%)	25 (54.3%)	*< 0.001*
Chronic renal disease	15 (14.7%)	6 (10.7%)	9 (19.6%)	0.209
Chronic liver disease	13 (12.7%)	9 (16.1%)	4 (8.7%)	0.266
COPD	10 (9.8%)	2 (3.6%)	8 (17.4%)	*0.020*
Solid cancer	27 (26.5%)	13 (23.2%)	14 (30.4%)	0.411
Hematological malignancy	14 (13.7%)	8 (14.3%)	6 (13%)	0.856
Solid organ transplantation	8 (7.8%)	5 (8.9%)	3 (6.5%)	0.653
Previous hospitalization	40 (39.2%)	21 (37.5%)	19 (41.3%)	0.695
Previous antibiotic therapy	58 (56.9%)	35 (62.5%)	23 (50%)	0.205
Length of ICU stay after KPC-Kp BSI, days, median (IQR)	18 (9–28.5)	24 (15.75–44.75)	12 (5–18)	*< 0.001*
ICU stay, days, median (IQR)	39 (21–59)	44.5 (29.75–75.5)	27 (15.5–50.75)	*< 0.001*
Hospital length of stay before bacteremia, days, median (IQR)	17.5 (5.75–36.5)	18.5 (7–34.25)	13 (3.75–39)	0.362
Primary cause of ICU admission	16 (15.7%)	8 (14.3%)	8 (17.4%)	0.668
Trauma	16 (15.7%)	11 (19.6%)	5 (10.9%)	0.225
Respiratory failure	16 (15.7%)	12 (21.4%)	4 (8.7%)	0.078
Cardiovascular disease	15 (14.7%)	6 (10.7%)	9 (19.6%)	0.209
Surgery	13 (12.7%)	7 (12.5%)	6 (13%)	0.935
Infection	11 (10.8%)	6 (10.7%)	5 (10.9%)	0.980
Burn injury	10 (9.8%)	3 (5.4%)	7 (15.2%)	0.096
Cerebrovascular accident	5 (4.9%)	3 (5.4%)	2 (4.3%)	0.814
Other*				
Source of infection				
CVC-related bacteremia	13 (12.7%)	11 (19.6%)	2 (4.3%)	*0.021*
Primary bacteremia	14 (13.7%)	2 (3.6%)	12 (26.1%)	*0.001*
Respiratory tract	11 (10.8%)	6 (10.7%)	5 (10.9%)	0.980
Urinary tract	21 (20.6%)	12 (21.4%)	9 (19.6%)	0.817
Skin and skin structure	18 (17.6%)	13 (23.2%)	5 (10.9%)	0.104
Intra-abdominal	24 (23.5%)	12 (21.4%)	12 (26.1%)	0.581
Endocarditis	1 (0.9%)	0	1 (2.2%)	0.268
Type of acquisition				
Healthcare-associated	13 (12.7%)	3 (5.4%)	10 (21.7%)	*0.014*
Nosocomial	89 (87.2%)	53 (94.6%)	36 (78.3%)	*0.014*
Charlson Comorbidity Index, median (IQR)	2 (1–3)	2 (0–3)	2 (1.75–3.25)	0.078
KPC-Kp intestinal colonization	52 (51%)	31 (55.4%)	21 (45.7%)	0.329
Source control	65 (63.7%)	40 (81.6%)	25 (80.6%)	0.912
Septic shock	40 (39.2%)	18 (32.1%)	22 (47.8%)	0.106
Mechanical ventilation	40 (39.2%)	23 (41.1%)	17 (37%)	0.672
AKI	15 (14.7%)	8 (14.3%)	7 (15.2%)	0.895
SOFA score, median (IQR)	5 (3–9.5)	4 (2–10)	7.5 (4–9.25)	*0.034*
APACHE II score, median (IQR)	15 (11–21)	13.5 (10–20)	19 (12–21)	*0.040*
DNR	3 (2.9%)	1 (1.8%)	2 (4.3%)	0.446
Time from blood culture collection to appropriate antibiotic therapy, hours, median (IQR)	15 (1–60)	8.5 (1–36)	48 (5–108)	*0.014*

*AKI* acute kidney injury, *BSI* bloodstream infection, *COPD* chronic obstructive pulmonary disease, *CVC* central venous catheter, *DNR* do not resuscitate order, *ICU* intensive care unit, *IQR* interquartile range, *KPC-Kp* KPC-producing *Klebsiella pneumoniae, SOFA* Sequential Organ Failure Assessment. Statistical significant *p* values are indicated in italics

* Other causes of ICU admission include 1 carbon monoxide poisoning, 1 Sezary syndrome, 1 acute renal failure, 1 Wilson disease with hepatic failure, 1 thyrotoxicosis

Susceptibility rates of the 102 KPC-Kp isolates are shown in Additional file 1: Table S1. The antibiotic regimens used for KPC-Kp BSIs in patients by survival status are shown in Additional file 1: Table S2. The most frequent antibiotic regimen was colistin plus meropenem plus tigecycline ± gentamycin (59.8%), followed by ceftazidime-avibactam alone or in combination with fosfomycin or aminoglycosides (12.7%) and other regimens (16.7%). Among 52 patients receiving a combination therapy including meropenem, MIC of the infecting strain was < 8 mg/L in 3.8% of cases, equal to 8 mg/L in 7.7%, ranging from 16 to 64 mg/L in 48.1%, and more than 64 mg/L in 40.4%. A prolonged infusion of antibiotics was used in 46 (88.5%) patients receiving meropenem and in 2 (15.4%) patients receiving CAZ-AVI. The proportion of patients not receiving in vitro active therapy was higher for those who died (19.6%) compared with those who survived (3.6%, *p* = 0.010). Two patients with CVC-related BSI not receiving any active antibiotic survived after a prompt removal of the infected device. Nine patients did not receive any antibiotic active in vitro and all of them died during the 30 days following bacteremia: 5 of them did not receive any antibiotic therapy because they died during the first 24 h from blood culture collection; 4 of them received a regimen containing colistin or tigecycline, but were affected by BSI due to pan drug-resistant Kp.

### Primary outcome analysis

The median time from blood culture collection to the start of appropriate antibiotic therapy was significantly shorter in surviving patients (8.5 h [IQR 1–36]) than in patients who died (48 h [IQR 5–108], *p* = 0.014). Among patients with previous rectal colonization by KPC-Kp, time from blood culture collection to appropriate antibiotic therapy was significantly shorter than that observed among non-colonized patients (5 h [IQR 0–49] vs 23.5 h [IQR 6–108.5], *p* = 0.004).

**Fig. 2:**
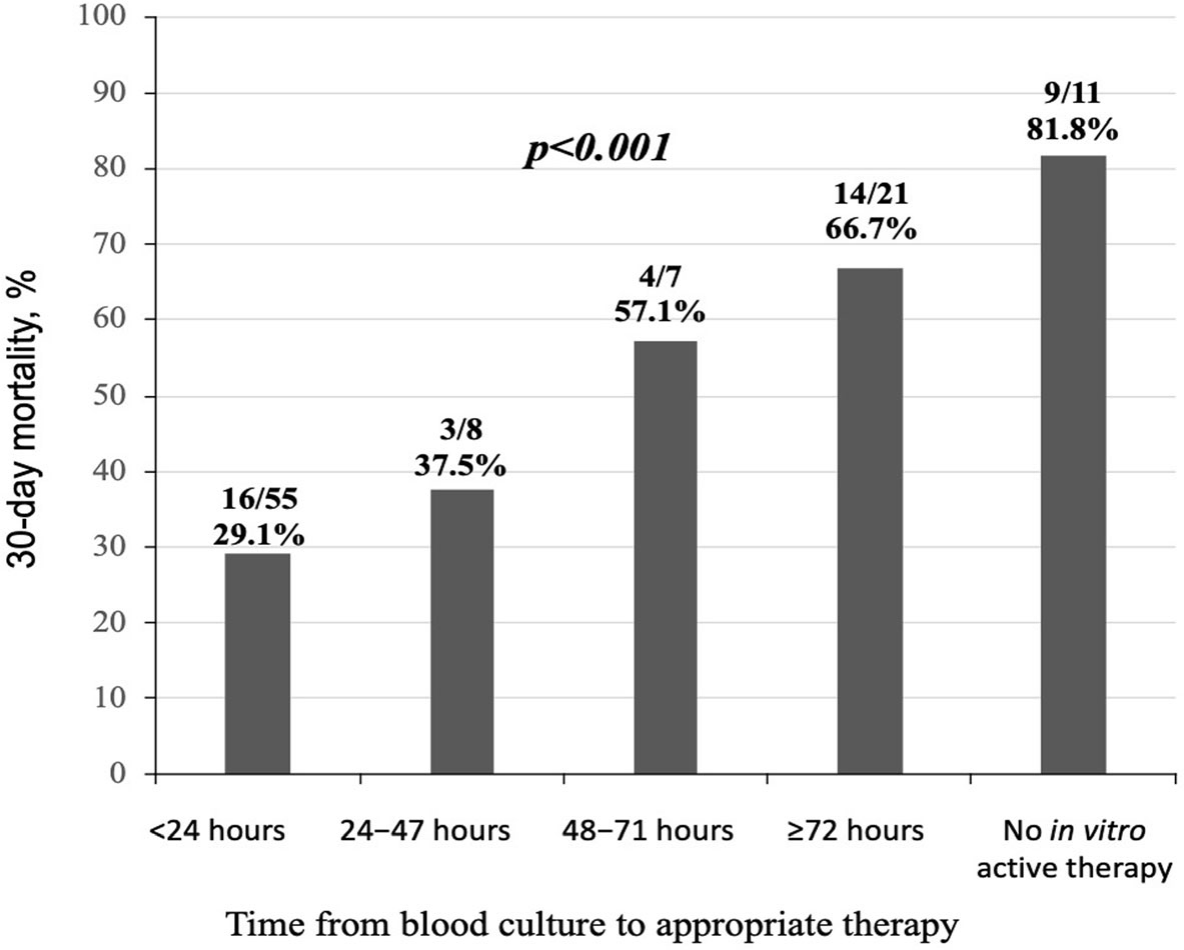
Thirty-day mortality rates by time from blood culture collection to appropriate antibiotic therapy

**Fig. 3:**
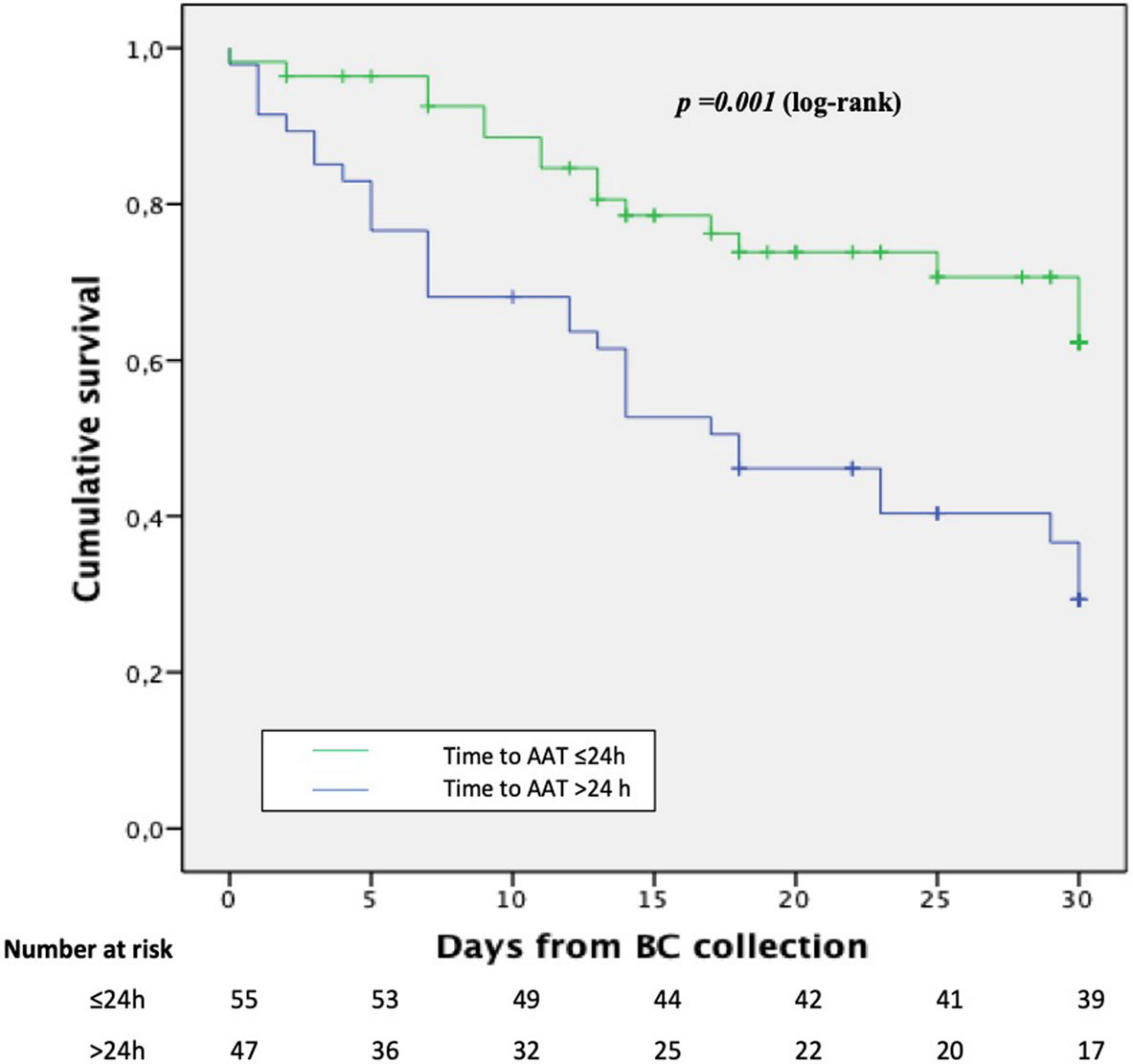
Kaplan-Meier for 30-day survival according to the receipt of appropriate antibiotic therapy within the first 24 h from blood culture collection. AAT appropriate antibiotic therapy; BC blood cultures

Figure 2 shows 30-day mortality rates according to different categories of time from blood culture collection to appropriate antibiotic therapy. Patients appropriately treated within the first 24 h (see Additional file 1: Table S3) had lower 30-day mortality rates (29.1% vs 63.8%, *p* < 0.001). Out of 52 patients receiving meropenem therapy, there were no significant differences in the distribution of MICs between patients receiving early (within 24 h) or late (> 24 h) appropriate therapy. After the exclusion of the 9 patients who did not receive any antibiotic active in vitro, 30-day mortality rates remained higher in patients who did not receive active antibiotic therapy compared to those who receive an appropriate antibiotic therapy within the first 24 h from the blood culture collection (58.3% versus 29.1%, *p* = 0.005). Figure 3 shows the Kaplan-Meier survival curves for patients who received or not an appropriate antibiotic therapy within 24 h from blood culture collection. A PS-based matched analysis was performed: we were able to match 47 couples to patients who received or not in vitro active therapy within 24 h from blood culture collection. The matched patients did not show significant differences in exposure to variables related to early antibiotic therapy. Conditional logistic regression in PS-matched cohorts showed that in vitro active therapy within 24 h from blood culture collection was associated with lower 30-day mortality (HR = 0.36, 95%CI: 0.188–0.690, *p* = 0.0021).

**Fig. 4:**
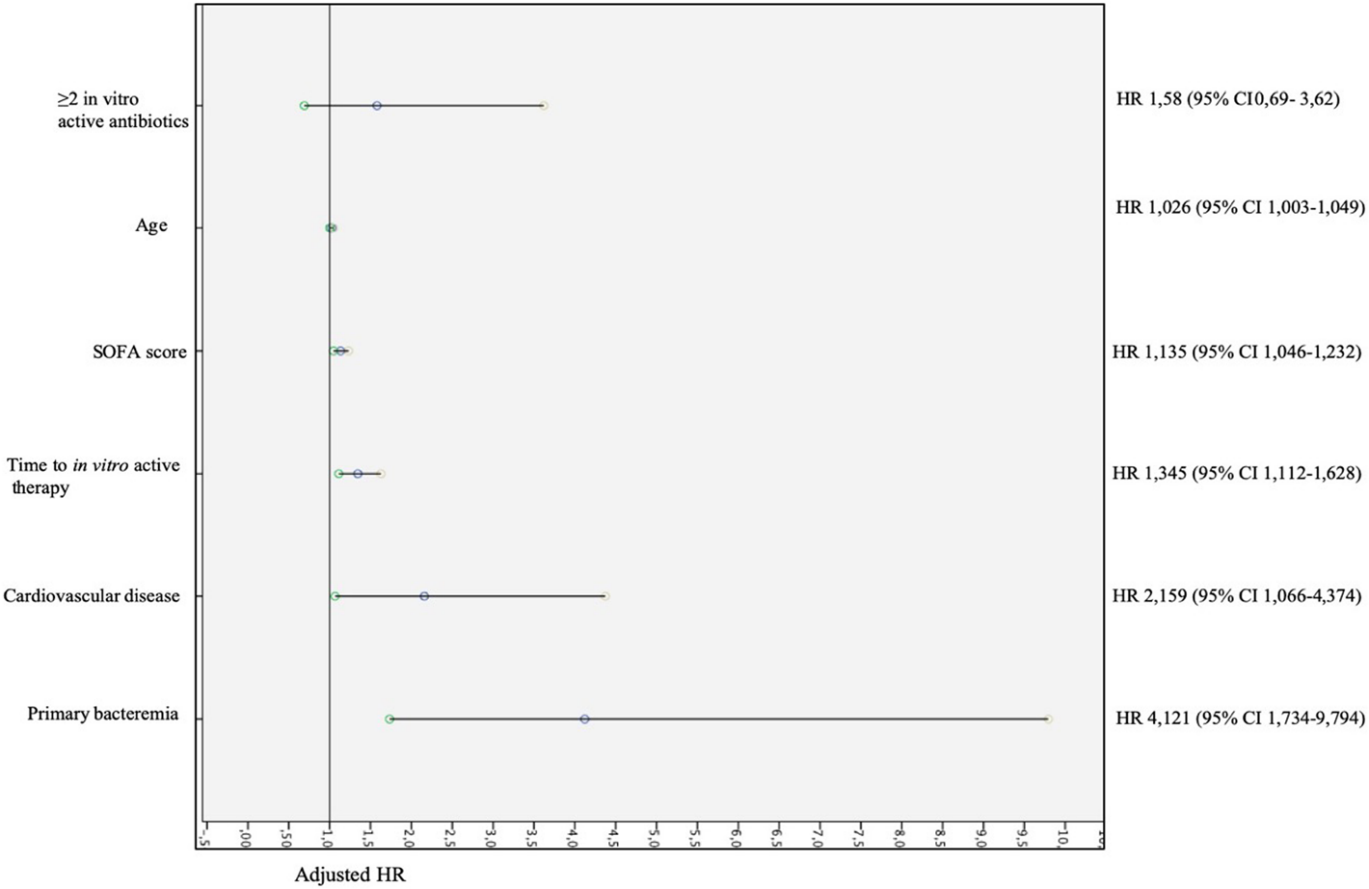
Forest plot showing adjusted HR of factors associated with 30-day mortality included in the sensitivity analysis

Factors independently associated with the overall 30-day mortality, by Cox regression analysis, were primary bacteremia (HR 2.662 [95% CI 1.118–6.336], *p* = 0.027) cardiovascular disease (HR 2.196 [95% CI 1.082–4.457], *p* = 0.029), time (24-h increments) from blood culture collection to appropriate therapy (HR 1.382 [95% CI 1.132–1.687], *p* = 0.001), SOFA score (HR 1.122 [95% CI 1.036–1.216], *p* = 0.005), and age (HR 1.030 [95% CI 1.006–1.054], *p* = 0.012) (Table 2). Time from blood culture collection to appropriate antibiotic therapy was independently associated with 30-day mortality, even excluding patients who received only tigecycline as active antibiotic. The sensitivity analysis showed that combination therapy with two or more in vitro active antibiotics was not associated with increased 30-day mortality (HR 1.58, 95% CI 0.69–3.62, *p* = 0.280). Figure 4 shows the forest plot of the adjusted HR of each factor included in the sensitivity analysis.

**Table 2:** Cox regression analysis of factors associated with 30-day mortality in patients with KPC-Kp BSI

	HR (95% CI)	*p* value
Primary bacteremia	2.662 (1.118–6.336)	*0.027*
Cardiovascular disease	2.196 (1.082–4.457)	*0.029*
Time from blood culture collection to appropriate therapy (24-h increments)	1.382 (1.132–1.687)	*0.001*
SOFA score (1-point increments)	1.122 (1.036–1.216)	*0.005*
Age (1-year increments)	1.030 (1.006–1.054)	*0.012*
Diabetes	1.960 (0.793–4.849)	0.145
COPD	1.303 (0.513–3.306)	0.578

*BSI* bloodstream infection, *CI* confidence interval, *HR* hazard ratio, *KPC-Kp* KPC-producing *Klebsiella pneumoniae*, *SOFA* Sequential Organ Failure Assessment. Statistical significant *p* value are indicated in italics

### Secondary outcome analysis

Table 3 shows the comparison of 30-day mortality and the composite endpoint according to treatment regimens. No treatment regimen influenced the 30-day mortality. Instead, CAZ-AVI-containing regimens were associated with reduced risk of composite endpoint (30-day mortality OR nephrotoxicity) (HR 0.231 [95% CI 0.071–0.745], *p* = 0.014) compared to colistin-containing regimens.

**Table 3:** Comparison of 30-day mortality and exploratory endpoint according to treatment regimens

	Antibiotic regimens	N (%)	HR (95%CI)	*p* value
30-day mortality				
	COL containing-regimen	27/61 (44.3%)	Ref variable	Ref variable
	CAZ-AVI containing-regimen	3/13 (23.1%)	0.424 (0.129–1.391)	0.157
	Other regimens	7/17 (41.2%)	0.799 (0.366–1.746)	0.574
Composite endpoint				
30-day mortality OR nephrotoxicity	COL containing-regimen	42/61 (68.9%)	Ref variable	Ref variable
	CAZ-AVI containing-regimen	3/13 (23.1%)	0.231 (0.071–0.745)	*0.014*
	Other regimens	8/17 (47.1%)	0.503 (0.245–1.034)	0.061

*COL* colistin*, CAZ-AVI* ceftazidime-avibactam, *Ref* reference. Statistical significant *p* value are indicated in italics

Additional file 1: Figure S1 shows the comparison of 30-day mortality among the three groups of antibiotic regimens stratified by site of infections. The higher 30-day mortality was observed in subjects with primary bacteremia (81.8%), followed by IAI (50%) and respiratory tract (33.3%), while the least mortality rate was observed in patients with CVC-related bacteremia (18%, *p* = 0.009 ANOVA test). In patients with primary bacteremia, mortality rate was higher in patients who received colistin-based regimen compared to those who received other antibiotic regimens (100% vs 50% *p* = 0.039). There were no differences in antibiotic dosages among patients with different site of infections.

## Discussion

This study demonstrates that time from blood culture collection to appropriate antibiotic therapy is independently associated with clinical outcome for ICU patients with BSI due to KPC-Kp, even after adjustment for comorbidities, severity of illness and age.

Delayed administration of appropriate antibiotic therapy is associated with high mortality rates in patients with sepsis or septic shock [29–32], and the probability of death increases with the number of hours of delay of antibiotic administration [31]. Moreover, time from blood culture collection to the administration of appropriate antibiotic therapy also influences the length of hospital stay [33]. No studies specifically evaluated the impact of the timing of appropriate antibiotic therapy in ICU patients with BSI by KPC-Kp. Our findings are unique in the population studied and could influence the future management of ICU patients with BSI due to KPC-Kp.

Our study demonstrated that the administration of appropriate antibiotic therapy within 24 h of blood culture collection is associated with 30-day mortality rates lower than 30%. Conversely, the risk of death progressively increased when appropriate antibiotic therapy was started 24–48 h (mortality rate 37.5%), 48–72 h (mortality rate 57.1%), and > 72 h (mortality rate 66.7%) from the index blood culture collection. Based on these results, the identification of patients with a high probability of BSI caused by KPC-Kp represents a great challenge for clinicians.

In the clinical practice, the median turnaround time from specimen collection to antimicrobial susceptibility testing results is 2.71 days [34]. In our study, this time is associated with increased mortality. Thus, it appears crucial to deliver effective therapy against KPC-Kp as soon as possible, and specifically within the first 24 h from the collection of blood cultures. A range of strategies may be useful in achieving this goal. According to the study results, our hospitals implemented the following recommendations for ICU patients: (i) regular rectal screening in all hospitalized patients to early identify KPC-Kp rectal carriers, (ii) starting an empirical therapy targeting KPC-Kp in rectal carriers with septic syndrome and contemporary presence of additional risk factors for KPC-Kp bacteremia (recent abdominal invasive procedure, receipt of chemotherapy/radiation therapy, additional colonization sites; a Giannella risk score ≥ 7 justify the administration of antibiotics covering KPC-Kp [35]), (iii) prompt de-escalation in case of not confirmed KPC-Kp infection as soon as microbiological results are available. This approach may be reasonable in endemic settings for KPC-Kp but cannot be generalized to other settings. New rapid diagnostic techniques enabling fast identification of genes coding for carbapenemase enzymes [36, 37] will be useful to improve this clinical strategy.

We found no differences in 30-day mortality rates among patients who received colistin-base, CAZ-AVI-based, or other treatment regimens. However, the composite endpoint of 30-day mortality or nephrotoxicity demonstrated a superior risk benefit profile for CAZ-AVI compared to colistin-containing regimens (*p* = 0.01). This is in line with recent reports showing that treatment-related nephrotoxicity was significantly higher in patients treated with colistin [38–40].

Furthermore, we were able to identify differences in mortality according to the site of infection. Patients with primary bacteremia and those with IAIs had the highest mortality rates, while patients with CVC-related BSI had the least mortality (81.8% vs 50% vs 18%, respectively *p* = 0.009); a large percentage of patients with primary bacteremia and IAI received colistin (63.6 and 37.5%, respectively) and the mortality in patients treated with colistin was higher than comparators. Although not statistically significant, this trend was observed also in patients with BSI arising from an IAI (64.3% vs 37.5%) and CVC-related bacteremia (37.5% vs 0%). A recent study showed that in patients with post-surgical IAI due to carbapenem-resistant *Acinetobacter baumannii* adjunctive colistin to conventional tigecycline did not yield clinical benefit but caused higher renal complication and unfavorable non-clinical outcomes [41]. It has been also documented that after the administration of a loading dose of colistin, concentration increases more slowly in peritoneal fluid than in plasma [42], and a reduced penetration rate and a potential inoculum effect of colistin in a difficult-to-reach site such as the abdominal cavity have been observed [43]. Conversely, despite the small number of cases, in patients with BSI from the urinary tract we observed a lower mortality in those who received colistin-containing regimens compared to other regimens (33.3% vs 100%). This may be due to a rapid urinary excretion and higher concentration in the urinary tract of intravenously administered colistin [44]. Unfortunately, the clinical efficacy of colistin therapy is difficult to assess because some routine laboratory methods used for colistin susceptibility testing (in particular gradient tests) have a poor performance [45], and is not ever possible to determine if colistin has appropriate MIC. Thus, reliable microbiological tests and colistin therapeutic drug monitoring should be considered to avoid toxicity and potential clinical consequences.

The observational nature of the study and the small sample size are intrinsic study limitations. Additionally, rather than the clinical diagnosis of BSI, we used blood culture collection time as the point from which to assess timing of appropriate antibiotic therapy, which may introduce a bias in the determination of the timing of appropriate therapy. This bias could potentially have been exacerbated if delays in obtaining blood cultures occurred after suspicion of infection. However, the choice of a well-defined time point allowed us to adopt an objective, standardized criterion. Although rapid identification methods were used, some patients received appropriate therapy after more than 48 h from BSI onset. It can be occurred because the time to blood culture positivity is variable and can range from few to several hours (or days). Moreover, in our institutions, the microbiology laboratories handle blood cultures 6/7 days weekly, and reporting may be delayed during weekend or public holidays; these factors could explain the prolonged time from blood culture collection to appropriate antibiotic therapy observed in some patients. Anyway, independently from the potential reasons underlining this delay, the time from blood culture collection and the appropriate antibiotic therapy was a factor associated with poor outcome. This finding highlights the crucial role of the microbiology laboratory and the importance of rapid reporting of microbiological results in the management of critically ill patients. Another limitation may be represented by the fact that we considered appropriate an antibiotic therapy if at least one antibiotic, including tigecycline, displayed in vitro activity. Considering its pharmacokinetic profile, the efficacy of tigecycline in patients with BSI due to KPC-Kp is debated and considering it an appropriate therapy may be questionable. However, in our study, we used a high dosage in all cases (100 mg every 12 h) and almost all published studies considered tigecycline as an evaluable and potentially effective drug [6]. Future studies should assess the exact role of tigecycline in KPC-Kp bacteremia.

## Conclusion

Our study shows that time from blood culture collection to appropriate therapy is an independent predictor of 30-day mortality in patients with KPC-Kp BSI. Clinicians should start appropriate antibiotic therapy as soon as possible, preferably within the first 24 h of blood culture collection. Treatment strategies allowing the early delivery of in vitro active antibiotics are urgently needed in ICU patients at risk of KPC-Kp bacteremia.

## Supplementary information

Additional file 1: **Table S1.** In vitro susceptibilities of KPC-producing blood isolates collected from patients hospitalized in ICU. **Table S2.** Definitive treatment regimens of patients by survival status after a KPC-Kp BSI. **Table S3.** Comparison between patients who received appropriate antibiotic therapy within the first 24 hours from the blood cultures collection and those who did not. **Figure S1.** Thirty-day mortality among patients who received different treatment regimens stratified by site of infection. (PDF 167 kb)

## Data Availability

The datasets used and/or analyzed during the current study are available from the corresponding author on reasonable request.

## Abbreviations

BSIs: Bloodstream infections
CI: Confidence interval
CR: Carbapenem-resistant
CVC: Central venous catheter
DNR: Do not resuscitate
ICU: Intensive care unit
IQRs: Interquartile ranges
Kp: *Klebsiella pneumoniae*
KPC: *Klebsiella pneumoniae* carbapenemase
SOFA: Sequential Organ Failure Assessment
