# Supplementary material for: Time to appropriate antibiotic therapy is a predictor of outcome in patients with bloodstream infection caused by KPC-producing Klebsiella pneumoniae
Source: Crit Care. 2020 Jan 30;24:29. doi: 10.1186/s13054-020-2742-9 (PMC6993311; doi:10.1186/s13054-020-2742-9)
Supplement: Supplementary file 1 — Table S1. In vitro susceptibilities of KPC-producing blood isolates collected from patients hospitalized in ICU. Table S2. Definitive treatment regimens of patients by survival status after a KPC-Kp BSI. Table S3. Comparison between patients who received appropriate antibiotic therapy within the first 24 hours from the blood cultures collection and those who did not. Figure S1. Thirty-day mortality among patients who received different treatment regimens stratified by site of infection. (PDF 167 kb) [file 13054_2020_2742_MOESM1_ESM.pdf]

Table S1. *In vitro* susceptibilities of KPC-producing blood isolates collected from patients hospitalized in ICU.

Bacterial species (isolate no.) and antimicrobial agent tested	Susceptibility rates (%)		
	S	I	R
Klebsiella pneumoniae (N=102)			
Ceftriaxone	-	-	100
Ceftazidime	-	-	100
Cefepime	-	-	100
PIP-TAZ	-	-	100
Ciprofloxacin	-	-	100
Levofloxacin	-	-	100
Amikacin	12.5	14.3	73.2
Gentamycin	42.9	30.4	26.8
Meropenem	-	2	98
Ertapenem	-	-	100
TMP-SMX	16.1	-	83.9
Tigecycline	61.5	9.6	28.8
Colistin	70.9	-	29.1
Fosfomycin	60.7	-	39.3

Table S2. Definitive treatment regimens of patients by survival status after a KPC-Kp BSI

	**All patients** ***N*=102**	**Patients who survived** ***n*=56**	**Patients who did not survive** ***n*=46**	***p* value**
No use of *in vitro* active antibiotics	11 (10.8%)	2 (3.6%)	9 (19.6%)	***0.010***
Only one *in vitro* active antibiotic used within 24 h	18 (17.6%)	13 (23.3%)	5 (10.9%)	0.104
Two or more *in vitro* active antibiotics used within 24 h	37 (36.3%)	26 (46.4%)	11 (23.9%)	***0.019***
Definitive therapy with fewer than two antibiotics displaying i*n vitro* activity	28 (27.4%)	18 (33.3%)	10 (27%)	0.522
Definitive therapy with two or more antibiotics displaying *in vitro* activity	63 (61.8%)	36 (66.7%)	27 (63%)	0.522
**Antibiotic regimens**				
Colistin-containing regimen	61 (59.8%)	34 (60.7%)	27 (58.7%)	0.836
CAZ-AVI ± aminoglycosides/fosfomycin	13 (12.7%)	10 (17.9%)	3 (6.5	0.088
Other regimens*	17 (16.7%)	10 (17.9%)	7 (15.2%)	0.722

BSI bloodstream infection, *CAZ AVI* ceftazidime-avibactam; *KPC-Kp* KPC-producing *Klebsiella pneumoniae*

* Other regimens include: 8 tigecycline + meropenem, 6 tigecycline + gentamycin, 3 meropenem+ertapenem

Table S3. Comparison between patients who received appropriate antibiotic therapy within the first 24 hours from the blood cultures collection and those who did not.

**Characteristic**	**Patients who received AAT <=24 hours N=55**	**Patients who did not received AAT <=24 hours N=47**	***p* value**
Age, years, median (IQR)	60 (53-71)	70 (53-77)	0.132
Male sex	34 (61.8%)	31 (66%)	0.665
Comorbidities			
Diabetes	9 (19.1%)	9 (16.4%)	0.713
Cardiovascular disease	13 (23.6%)	23 (48.9%)	0.008
Chronic renal disease	9 (16.4%)	6 (12.8%)	0.609
Chronic liver disease	9 (16.4%)	4 (8.5%)	0.236
COPD	3 (5.5%)	7 (14.9%)	0.110
Solid cancer	13 (27.7%)	14 (25.5%)	0.801
Hematological malignancy	9 (16.4%)	5 (10.6%)	0.402
Solid organ transplantation	5 (9.1%)	3 (6.4%)	0.612
Primary reason for ICU admission			
Trauma	8 (14.5%)	8 (17%)	0.732
Respiratory failure	7 (12.7%)	9 (19.1%)	0.374
Cardiovascular disease	9 (16.4%)	7 (14.9%)	0.839
Surgery	10 (18.2%)	5 (10.6%)	0.284
Infection	8 (14.5%)	5 (10.6%)	0.555
Burn injury	5 (9.1%)	6 (12.8%)	0.551
Cerebrovascular accident	3 (5.5%)	7 (14.9%)	0.110
Other*	5 (9.1%)	0	***0.040***
Previous hospitalization	28 (50.9%)	12 (25.5%)	***0.009***
Previous antibiotic therapy	34 (61.8%)	24 (51.1%)	0.274
Length of ICU stay after KPC-Kp BSI, days, median (IQR)	20 (13-30)	17 (7-25)	0.095
ICU stay, days, median (IQR)	40.5 (25.75-59.25)	33 (16.75-54.75)	0.074
Hospital length of stay before bacteremia, days, median (IQR)	19 (9-36)	14 (3-41)	0.215
Source of infection			
CVC-related bacteremia	10 (18.2%)	3 (6.4%)	0.075
Primary bacteremia	6 (10.9%)	8 (17%)	0.371
Respiratory tract	5 (9.1%)	6 (12.8%)	0.551
Urinary tract	13 (23.6%)	8 (17%)	0.410
Skin and skin structure	12 (21.8%)	6 (12.8%)	0.232
Intra-abdominal	9 (16.4%)	15 (31.9%)	0.065
Endocarditis	0	1 (2.1%)	0.277
Type of acquisition			
Healthcare-associated	2 (3.6%)	8 (17%)	***0.023***
Nosocomial	52 (94.5%)	37 (78.7%)	***0.017***
Charlson Comorbidity Index, median (IQR)	2 (1-4)	2 (1-3)	*0.305*
KPC-Kp intestinal colonization	33 (60%)	19 (40.4%)	***0.049***
Source control	39 (70.9%)	26 (55.3%)	0.103
Septic shock	23 (41.8%)	17 (36.2%)	0.560
Mechanical ventilation	26 (47.3%)	14 (29.8%)	0.071
AKI	9 (16.4%)	6 (12.8%)	0.609
SOFA score, median (IQR)	6 (3-10)	5 (2-9)	0.355
APACHE II score, median (IQR)	17 (12-22)	15 (11-21)	0.302
DNR	1 (1.8%)	2 (4.3%)	0.468

Overall 30-day mortality	16 (29.1%)	30 (63.8%)	***<0.001***
Thirty-day mortality attributable to infection	15 (27.3%)	28 (59.6%)	***0.001***
Overall 14-day mortality	11 (20%)	22 (46.8%)	***0.004***
Overall 7-day mortality	6 (10.9%)	15 (31.9%)	***0.009***

AAT appropriate antibiotic therapy*, AKI* acute kidney injury, *BSI* bloodstream infection, *COPD* chronic obstructive pulmonary disease, *CVC* central venous catheter, *DNR* do not resuscitate order, *ICU* intensive care unit, *IQR* interquartile range, *KPC-Kp* KPC-producing *Klebsiella pneumoniae, SOFA* Sequential Organ Failure Assessment

* Other causes of ICU admission include: 1 carbon monoxide poisoning, 1 Sezary syndrome, 1 acute renal failure, 1 Wilson disease with hepatic failure, 1 thyrotoxicosis.

Figure S1. Thirty-day mortality among patients who received different treatment regimens stratified by site of infection.

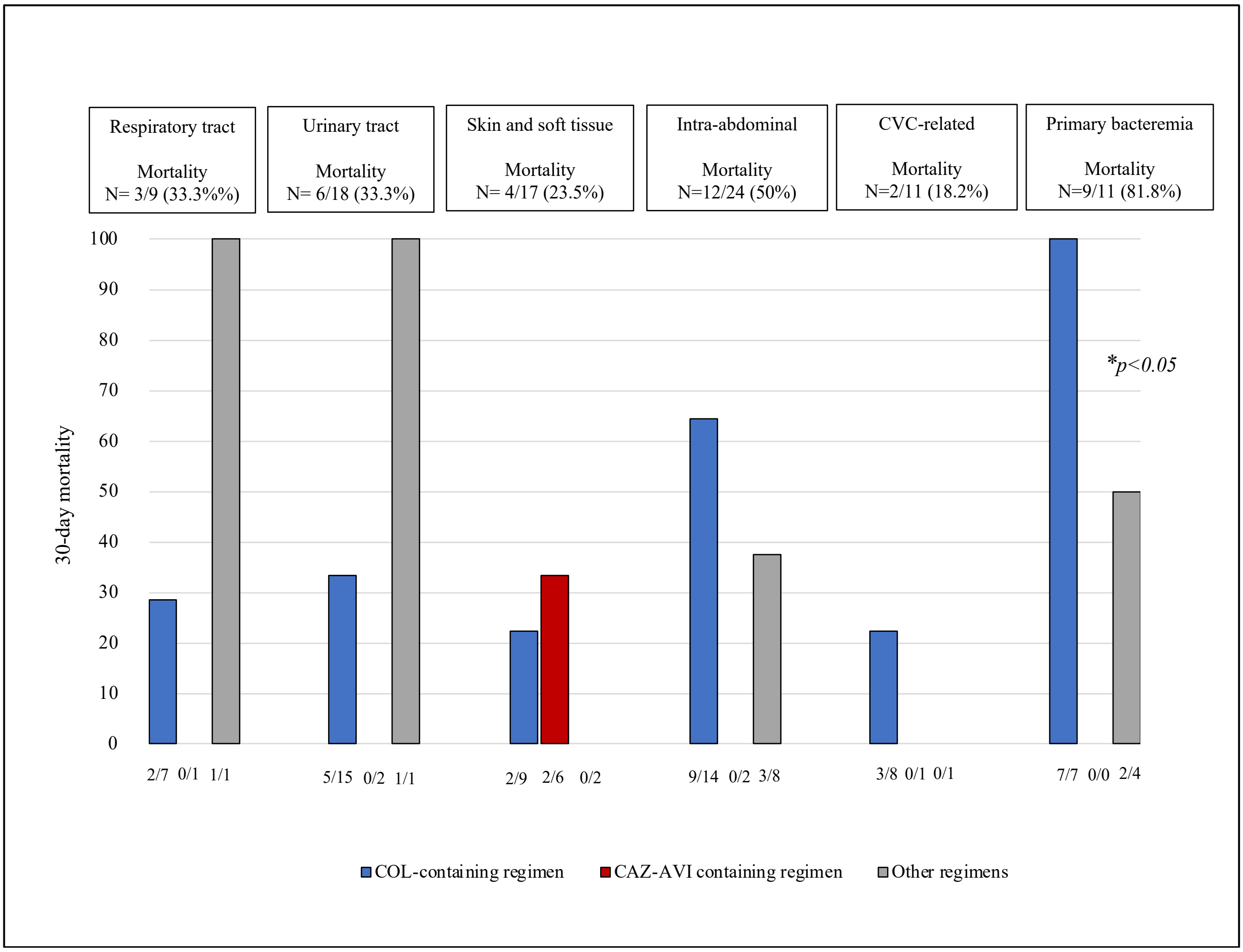

Patients who did not received in vitro active therapy (N=9) were excluded.
One patient with endocarditis treated with ceftazidime/avibactam died (not shown in the Figure).
COL= colistin; CAZ-AVI= ceftazidime-avibactam
